# Genome sequence and characterization of the *bcs* clusters for the production of nanocellulose from the low pH resistant strain *Komagataeibacter medellinensis* ID13488

**DOI:** 10.1111/1751-7915.13376

**Published:** 2019-02-22

**Authors:** Ana M. Hernández‐Arriaga, Carlos del Cerro, Leire Urbina, Arantxa Eceiza, Mª Angeles Corcuera, Aloña Retegi, M. Auxiliadora Prieto

**Affiliations:** ^1^ Polymer Biotechnology Group Microbial and Plant Biotechnology Department Centro de Investigaciones Biológicas CIB‐CSIC Ramiro de Maeztu 9 28040 Madrid Spain; ^2^ Materials + Technology’ Group Engineering School of Gipuzkoa Department of Chemical and Environmental Engineering University of the Basque Country (UPV/EHU) Pza. Europa 1 20018 Donostia – San Sebastián Spain; ^3^ Present address: National Bioenergy Center National Renewable Energy Laboratory 15013 Denver West Parkway Golden CO 80403 USA

## Abstract

*Komagataeibacter medellinensis* ID13488 (formerly *Gluconacetobacter medellinensis* ID13488) is able to produce crystalline bacterial cellulose (BC) under high acidic growth conditions. These abilities make this strain desirable for industrial BC production from acidic residues (e.g. wastes generated from cider production). To explore the molecular bases of the BC biosynthesis in this bacterium, the genome has been sequenced revealing a sequence of 3.4 Mb containing three putative plasmids of 38.1 kb (pKM01), 4.3 kb (pKM02) and 3.3 Kb (pKM03). Genome comparison analyses of *K. medellinensis* ID13488 with other cellulose‐producing related strains resulted in the identification of the *bcs* genes involved in the cellulose biosynthesis. Genes arrangement and composition of four *bcs* clusters (*bcs1, bcs2, bcs3* and *bcs4*) was studied by RT‐PCR, and their organization in four operons transcribed as four independent polycistronic mRNAs was determined. qRT‐PCR experiments demonstrated that mostly *bcs1* and *bcs4* are expressed under BC production conditions, suggesting that these operons direct the synthesis of BC. Genomic differences with the close related strain *K. medellinensis* NBRC 3288 unable to produce BC were also described and discussed.

## Introduction

Bacterial cellulose (BC) is an organic polymer produced by certain types of bacteria. This biopolymer has been proposed as a new biomaterial due to its unique physical, mechanical, chemical and structural properties (Jozala *et al*., 2016). Bacterial cellulose features like the absence of impurities (such as hemicelluloses, lignin, pectin and wax) or a synthesis that relies on the production of continuous interconnected fibres (Lee *et al*., 2014), make this polymer more desirable for some specific applications in comparison with plant cellulose (Jonas and Farah, 1998; Lee *et al*., 2014; Jozala *et al*., 2016). Thus, BC has been used in biomedical and pharmaceutical applications, in food industry, as well as in other industrial applications such as acoustics, electronic paper displays or as a reinforcement agent for gels and films (Chawla *et al*., 2009).

In bacteria, amorphous cellulose is present in the extracellular matrix component of biofilms. Aside from its structural properties, other specific biological roles of the BC are related with the maintenance of aerobic environments, flocculation processes or plant attachment mechanisms (Lee *et al*., 2014; Augimeri *et al*., 2015; Jozala *et al*., 2016). The production of this polymer has been reported among a great variety of bacterial genus including some *Acetobacteraceae* species, plant symbionts such as *Rhizobium* (Ausmees *et al*., 1999), soil bacteria such as *Pseudomonas* (Ude *et al*., 2006) as well as plant pathogens including *Dickeya* (Jahn *et al*., 2011) and *Agrobacterium* (Matthysse *et al*., 1995). Even human gut microbes such as *Escherichia coli* or *Salmonella enterica* are capable of producing cellulose (Zogaj *et al*., 2003). Production of BC also has been detected in some Gram‐positive bacteria including *Sarcina ventriculi* (Ross *et al*., 1991).

**Figure 1:**
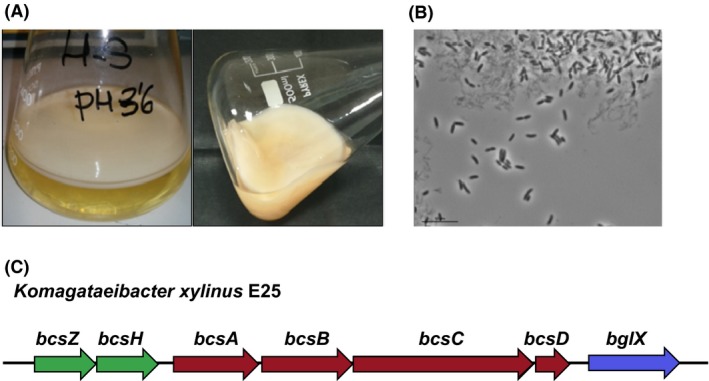
A. Cellulose produced by *K. medellinensis* ID13488. Cells were grown in HS (1X) standard media, pH 3.6, in static conditions for 14 days. The cellulose mat over the liquid culture can be observed (left and right panel images).B. Optical microscope images of *K. medellinensis* ID13488 cells producing cellulose. Cultures were grown as described above. Bacteria can be observed free or attached to the cellulose in the liquid media culture.C. Genetic map of the *bcs* cluster in the model strain *K. xylinus* E25. Genes (represented as arrows) essential for cellulose synthesis *in vivo* (*bcsA*,* bcsB*,* bcsC* and *bcsD*) are coloured in orange. Accessories genes are coloured in blue (*bglX*) or green (*bcsZ* and *bcsH*). The *bcsA* and *bcsB* gene products constitute the cellulose synthase subunit. The product of the *bcsC* and *bcsD* genes are proteins involved in the secretion and crystallization of the glucan chain. The products of *bcsZ* and *bglX* has been identify as an endoglucanase and a β‐glucosidase, respectively. *bcsH* encodes a protein involved in the arrangement of glucan chains into crystalline cellulose.

The most effective cellulose‐producing bacteria belong to the *Gluconacetobacter* and *Komagataeibacter* genus, included in the *Acetobacteraceae* family. Some *Komagataeibacter* species are able to produce two forms of cellulose, designated as cellulose I and cellulose II, which have different microfibrillar structures (Chawla *et al*., 2009; Matsutani *et al*., 2015). Cellulose I consists of a ribbon‐like polymer (crystalline cellulose), while cellulose II is an amorphous polymer. The difference in the structural assembly of cellulose I and II microfibrils seems to rely on the composition of the bacterial cellulose synthase enzymatic complex (BCS complex). Up to now, two types (type I and type II) of *bcs* operons responsible for the synthesis of cellulose in *Komagataeibacter* species have been reported (Umeda *et al*., 1999; Romling and Galperin, 2015). The type I operon (*bcs1* operon), first identified in the prototypic strain *K. xylinus* E25 (Saxena *et al*., 1990; Wong *et al*., 1990), is a four‐gene *bcs*ABCD operon encoding BcsA1, BcsB1, BcsC1 and BcsD1 proteins (Fig. 1). The four proteins are required for a maximum cellulose production *in vivo* (Wong *et al*., 1990). Proteins BcsA1 and BcsB1 constitute the BCS complex glycosyl transferase catalytic subunit. These proteins transfer glucosyl residues from UDP‐glucose to the β‐D‐1,4‐glucan chain and *in vitro* assays proved that they are the only proteins required for cellulose synthesis (Omadjela *et al*., 2013; Morgan *et al*., 2014). BcsC1 and BcsD1 are involved in exporting and packing the polymer fibrils at the cell surface by forming a channel into the outer membrane that allows crystallization of BC (Wong *et al*., 1990; Hu *et al*., 2010; Metha *et al*., 2015). In addition to this, other genes with functions related with the cellulose production are commonly found in *bcs* type I operons. Some examples are *bcsZ*, encoding an endoglucanase, *bglX*, encoding a β‐glucosidase or *bcsH* which is required for the cellulose production affecting the expression levels of *bcsB* and *bcsC* (Deng *et al*., 2013). BcsH has also been proposed to be involved in the arrangement of the polymer chains into crystalline ribbons (Standal *et al*., 1994; Deng *et al*., 2013; Sunagawa *et al*., 2013). However, some exceptions have been reported in the type I operon arrangement. For example, *K. hansenii* ATCC 23769 and *K. swingsii* ATCC 5358 (Saxena *et al*., 1994; Saxena and Brown, 1995) have the *bcsA1* and *bcsB1* genes fused together encoding a single catalytic subunit called BcsAB1 and the type II operon (*bcs2*) could be also constituted by a fused gene (*bcsAB2)*,* bcsC2* and two extra genes, *bcsX* and *bcsY* (Umeda *et al*., 1999; Nakai *et al*., 2013). Although the product of these genes has not been characterized, *in silico* sequence comparisons indicate a putative transacylase function for BcsY suggesting that BcsY is probably involved in the production of acetylcellulose or other similar polysaccharide (Umeda *et al*., 1999; Chawla *et al*., 2009). BcsX has been proposed as a cellulose deacylase (Umeda *et al*., 1999).

Recently, the complete genome sequence of several *Komagataeibacter* species has been reported (Ogino *et al*., 2011; Kubiak *et al*., 2014; Florea *et al*., 2016b; Zhang *et al*., 2017; Ryngajłło *et al*., 2018). The genomic data analyses of these cellulose‐producing bacteria showed a great diversity in *bcs* operons. Thus, in contrast to *K. xylinus* E25 that encodes two *bcs* operons, up to four operons *bcs1*,* bcs2*,* bcs3* and *bcs4*, which differ in their structure and genetic organization have been described in the genomes of *K. medellinensis* NBRC 3288 (natural non‐cellulose producer strain) (Ogino *et al*., 2011; Matsutani *et al*., 2015), *G. hansenii* ATCC 53582 (Florea *et al*., 2016b) *K. rhaeticus* iGEM (Florea *et al*., 2016a) and *G. xylinus* CGMCC 2955 (Liu *et al*., 2018). Although the genome sequences of these related strains have become available, and their *bcs* cluster coding regions have been elucidated, the organization of the *bcs* transcription units needs to be explored experimentally.

Among BC‐producing strains, *K. medellinensis* ID13488 has also been isolated and characterized for its ability to produce cellulose in high acidic growth conditions [pH 3.5 (Castro *et al*., 2012, 2013)] (Fig. 1). Thus, ID13488 is able to produce 4.5 g l^−1^ of cellulose at this pH value using 2% glucose as carbon source in the standard HS growth media (Castro *et al*., 2012). Recently, the production of BC by this strain using different carbon sources or even using by‐products of the cider industry has also been reported (Molina‐Ramírez *et al*., 2017; Urbina *et al*., 2017), making strain ID13488 suitable for particular industrial applications involving the use of acidic residues as feedstock.

In this work, we have sequenced and analysed the genome of *K. medellinenis* ID13488 strain. We have characterized by RT‐PCR assays the operon organization of the four *bcs* clusters identified and have evaluated the expression of each operon at BC production conditions.

## Results and discussion

### Genome sequencing and annotation

*K. medellinensis* strain ID13488 genome was sequenced to approximately 251‐fold coverage using PacBio technologies, and after a *de novo* assembly, a sequence of 3.4 Mbp in size was obtained with a 60.7% of GC Content. The resulting assembly was organized in three contigs and only one circular sequence of 38 kbp with a %GC of 43.9. Assembled sequences were automatically annotated using the Rast web‐tool pipeline obtaining a total of 3426 coding sequences and 73 RNAs. The functional landscape of the strain ID13488 was categorized following the Rast subsystem catalogue. The highest number functional hits were found in the categories related with, ‘amino acids and derivatives’, ‘protein metabolism’ and ‘carbohydrates’, in this order (Fig. S1).

Additionally, to determine the existence of plasmids in the strain ID13488, plasmid DNA was isolated and sequenced using Illumina Miseq technologies. Resulting reads were assembled, and circular sequences corresponding to three putative plasmids were obtained. The longest sequence of 38 059 bp coincided exactly with the only circular sequence obtained from the PacBio assembly and was designated as pKM01. The other two circular sequences of 4289 and 3314 bp were named pKM02 and pKM03, respectively. The three putative plasmids sequences were also annotated using Rast and 61 putative ORFs were detected for pKM01, eight for pKM02 and four for pKM03. Almost all the functional annotations obtained for the three sequences resulted in hypothetical proteins. To reveal additional functional information, Pfam domain searches were performed. For pKM01, several functional domains related with viral proteins were found and almost all of them were located in only one strand. This fact and the very different GC content (43.9%) suggest a possible bacteriophage‐related origin of the sequence. Several domains usually found in other plasmids MobA/MobL (PF03389) and ParE (PF05016) were detected in pKM02, and no functional domains were identified in pKM03. Putative plasmids were also compared against other *Komagataeibacter* sequences at the NCBI nucleotides database. Partial conserved blast hits (95% identity and 40% of query coverage) were obtained for pKM01, only against *Komagataeibacter nataicola* strain RZ201 pKNA05 plasmid sequence (CP019880). Similar sequences to pKM02 were detected in *K. medellinensis* NBRC 3288 (plasmid pGXY060, AP012165) (88% identity and 79% of query coverage) and *K. xylinus* E25 (plasmid pGX1, CP004361) (86% identity and 69% of query coverage). No significative blast hits were obtained for pKM03, suggesting that this is a plasmid exclusively found in the strain ID13488.

### Phylogenetic analyses and *in silico* strain comparisons

**Figure 2:**
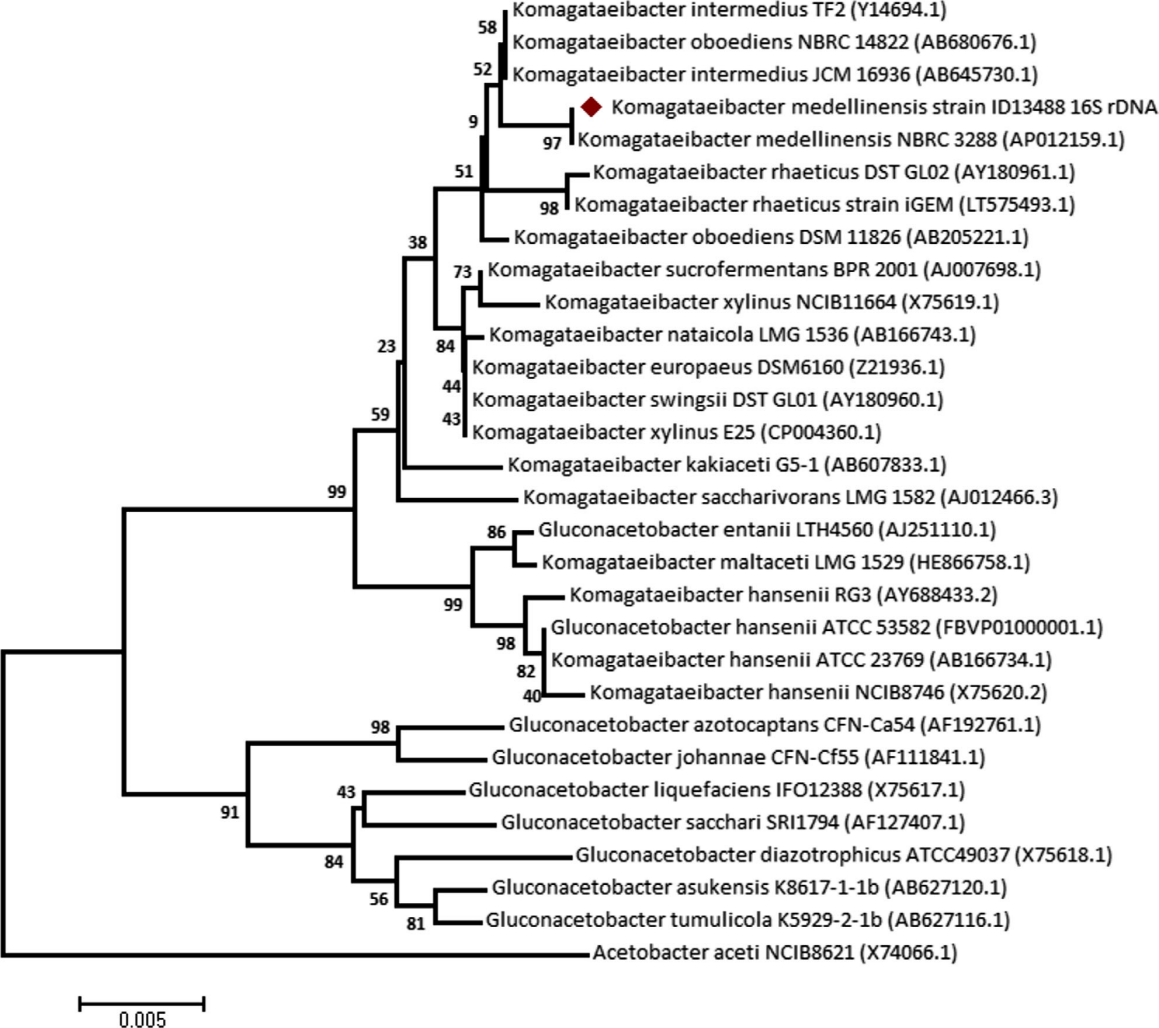
16S rRNA phylogenetic tree of *K. medellinensis* strain ID13488 and other related strains. The tree was built using the Neighbour‐Joining method with bootstrap test values (from 1000 replicate) expressed as a percentage of 100 at the branch points. A red mark indicates the position of the strain ID13488 in the tree. Genebank accession numbers are shown in parenthesis. *Acetobacter aceti* NCIB8621 16s rRNA was used as outgroup.

16S rRNA gene was detected in *K*. *medellinensis* strain ID13488 sequence, obtaining a total of five exact copies of this gene distributed in different *loci* of the genome. This DNA sequence of 1486 bp was compared with other 16S rRNA gene sequences belonging to related and selected type strains. All the selected sequences were aligned, and a phylogenetic tree was generated (Fig. 2). Two main clades can be observed in the phylogenetic tree: one containing mainly *Komagataeibacter* sequences and other that groups all the *Gluconacetobacte*r strains except *G. hansenii* ATCC 53582 and the *G. entanii* LTH4560. This clade containing the *G. hansenii* species was also observed in Florea *et al*. (2016b) and Ryngajłło *et al*. (2018). This fact suggests that these strains could be taxonomically assigned to the *Komagataeibacter* genus instead of the *Gluconacetobacter*. The strain ID13488 that was classified previously as a *Gluconacetobacter,* appears included in the *Komagataeibacter* clade and for this reason, this strain will be named as *Komatagaeibacter medellinensis* strain ID13488 from hereafter. The most similar sequence to the strain ID13488 16S rRNA that belonged to *K. medellinensis* strain NBRC 3288 and appears clustered with the strain ID13488. The 16S rRNA gene of both strains resulted identical.

To confirm the information given by the 16S rRNA analyses, whole genome sequence comparisons were performed including the strain ID13488 and other four related strains of interest (*K. medellinensis* NBRC 3288, *K. rhaeticus* iGEM, *K. hansenii* ATCC23769 and *K. xylinus* E25). ANI (average nucleotide identities) values were calculated for all these strains (Table S1). The high ANI values found between *K. medellinensis* strain ID13488 and NBRC 3288 (98.86%) corroborate that both strains could be considered as a single species (Richter and Rossello‐Mora, 2009). The strain ID13488 seems to be more related with the cellulose producer *K. rhaeticus* (88.20%) than *K. xylinus* E25 (85.89%) and *G. hansenii* ATCC23769 (85.2%). Calculated tetra‐nucleotide frequencies (Table S1) between all these strains confirm these facts showing a high correlation between the strains ID13488 and NBRC 3288 (0.999), lower values for ID133488 compared with *K. rhaeticus* iGEM and *K. xylinus* E25 (0.970 and 0.955), and a correlation coefficient against *G. hansenii* ATCC23769 (0.813) that suggest again that these two strains are more distant than the rest (Richter and Rossello‐Mora, 2009).

### Identification and *in silico* analysis of the *K. medellinenis* bcs clusters

**Figure 3:**
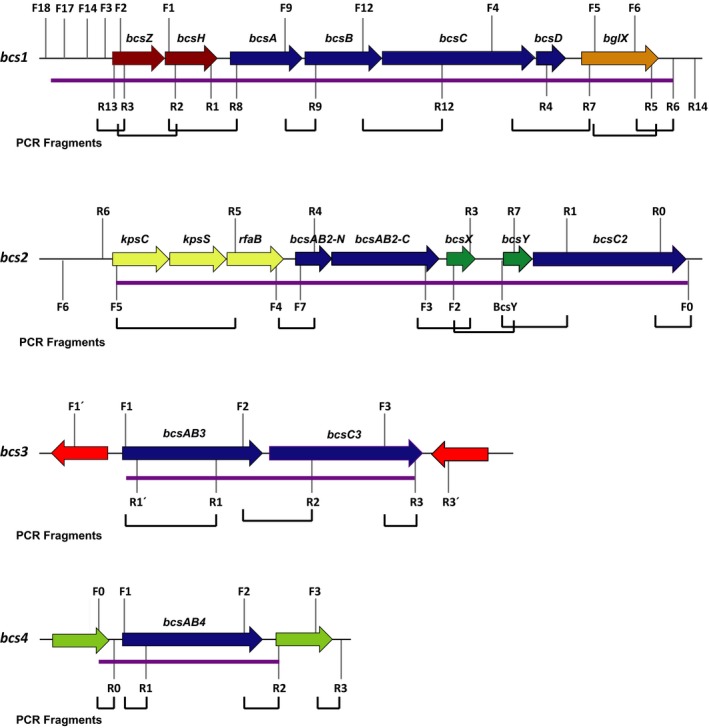
*K. medellinensis* strain ID13488 *bcs* clusters. Genetic organization of the four *bcs* clusters identified in the strain ID13488. Arrows indicate each one of the genes found in the cluster. Genes required for the cellulose production appear in blue and accessory genes appear in other different colours. Numbered letters indicate the position of the primers employed for each cluster in the RT‐PCR assays and the brackets indicate the DNA fragments that were amplified. The *bcs1* operon encodes BcsA and BcsB which constitute the cellulose synthase subunits of the BCS complex; BcsC and BcsD that are involved in the translocation and crystallinity of the glucan chains; and BcsZ and BglX that have been identified as an endoglucanase and a β‐glucosidase, respectively. The *bcs2* operon encodes the fused BcsAB2 and BcsC2 cellulose synthase and translocase subunits; KpsC, KpsS and RfaB, proteins associated with extracellular matrix formation; and BcsY and BcsX that have been predicted as a transacylase and deacylase proteins. BcsAB3 and BcsC3 constitute the synthase and translocase BCS subunits encoded by *bcs3* operon and BcsAB4 fused protein is the synthase subunit encoded by *bcs4* operon. Purple bars indicate the genes and the DNA regions that have been tested for each operon.

A total of four putative *bcs* clusters (*bcs1*,* bcs2, bcs3* and *bcs4*) have been identified in the genome of *K. medellinensis* strain ID13488 by *in silico* analyses. All four clusters show a different gene composition and structure among them (Fig. 3). The *bcs1* cluster comprises seven ORFs corresponding to *bcsZ*,* bcsH*,* bcsA*,* bcsB, bcsC*,* bcsD* and *bglX*. The genes *bcsZ* and *bcsH* are located 186 bp upstream of *bcsA* whereas *bglX* is 180 bp downstream of *bcsD*. Genes *bscA* to *bcsD* are physically adjacent in the genome comprising a total of 13 795 bp.

**Figure 4:**
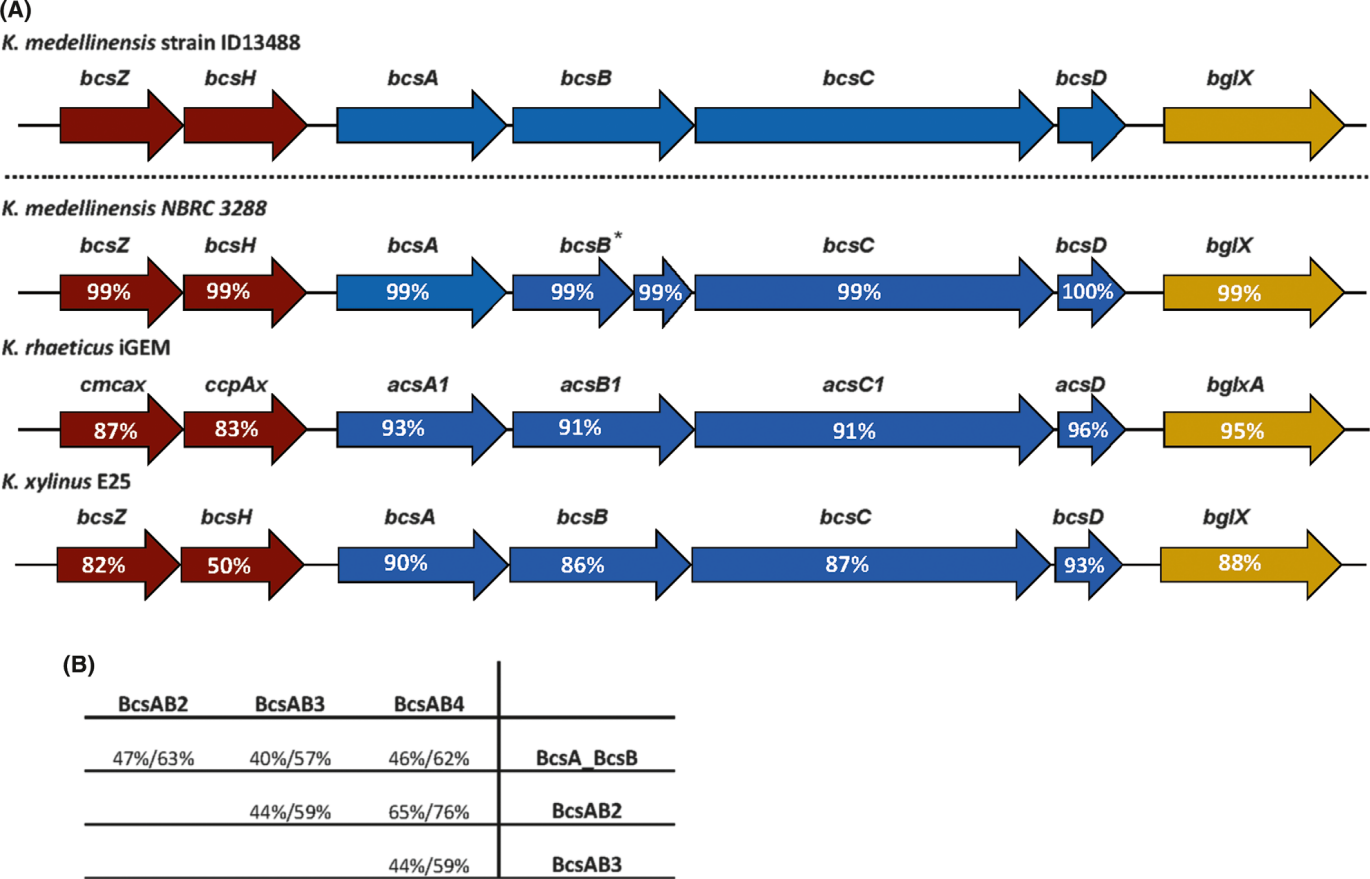
*bcs1* cluster comparison of *K. medellinensis* strain ID13488 with related strains.A. Genes (represented as arrows) contained in the *bcs1* cluster of four related strains were compared against the ones contained in the strain ID13488. Pairwise blast was used to estimate the distance between homologous genes. The identity percentage values are given inside the arrows. The asterisk points a premature STOP codon in the *bcsB* ORF for the strain NBRC 3288.B. Pairwise blast analyses were performed between all the cellulose synthase genes. Identity (left) and positives (right) percentages are given for each one of the comparisons. To perform the analyses, *bcsA* and *bcsB* genes from the *bcs1* operon were combined as a unique amino acid sequence.

Several *bcs* clusters have also been detected in other known cellulose‐producing species as *K. xylinus* E25, *K. hansenii* ATCC 53582, *K. rhaeticus* IGEM or *G. xylinus* CGMCC 2955 (Kubiak *et al*., 2014; Florea *et al*., 2016a,b; Liu *et al*., 2018). The detected *bcs* clusters (*bcs1*,* bcs2*,* bcs3* and *bcs4*) in the genome of *K. medellinensis* ID13488 display a very similar organization to that described in the *K. rhaeticus* iGEM genome (Florea *et al*., 2016a). As shown for *bcs1* in *K. rhaeticus*,* bcsA* and *bcsB* are also presented in separated ORFs, and a single copy of *bcsD* has been detected. The *bcs1* cluster also contains other putative cellulose production‐related genes surrounding the main *bcsABCD* genes. These genes are an endoglucanase coding gene similar to the *bcs*Z gene from *Komagateibacter* species; *bcsH* that encodes a protein that affects expression levels of *bcsB* and *bcsC*, and *bglX* that putatively encodes β‐glucosidase enzyme (Fig. 3). A comparison of the cluster *bcs1* with other clusters with similar structures is shown (Fig. 4). The pair‐blast high identity values indicated that, in general, the proteins are very conserved among these different species. One of the main exceptions to this fact is a deletion of 18 bp and a premature codon stop in *bcsB1* that was described in the strain *K. medellinensis* NBRC 3288 (Ogino *et al*., 2011; Matsutani *et al*., 2015) responsible for the non‐producing BC phenotype of this strain.

Concerning the *bcs2* cluster, five ORFs related with cellulose biosynthesis have been identified. A long fused *bcsAB2* (which comprises *bcsAB2*‐N and *bcsAB2*‐C ORFs, see below), is followed 110 bp downstream by *bcsX*. Finally*,* the *bcsC2* gene is located at the end of the *bcs* cluster. Upstream of *bcsAB2*‐N three genes, *kpsC*,* kpsS* and *rfaB*, which are separated 588 bp upstream to *bcsAB2*‐N, were detected (Fig. 3). The sequence analysis of *bcs2* cluster showed a truncated long fusion gene *bcsAB2* containing a premature stop codon that differs from other *Komagateibacter* species. This phenomenon is the result of the disruption of this gene generating two different open reading frames: *bcsAB2*‐N of 996 bp and *bcsAB2‐*C of 3564 bp. The propagation of an identified repeated sequence that could be described as a microsatellite seems to be the cause of the appearance of this stop codon. Although this repeated pattern might be found in other non‐truncated versions of this gene in other related species such as the strain NBRC 3288, the ID13488 version contains extra repetitions generating a stop codon which seems to be characteristic of the strain ID13488. However, *bcsAB2*‐C contains the entire BcsB (PF03170) domain suggesting that this ORF could still be producing an active short version of the enzyme. Additionally, a *bcsC2* copy that presents differences at the initial region with the strain NBRC 3288 copy of this gene and other genes related with the extracellular matrix formation (*kpsC*,* kpsS* and *rfa*B) were found in the 5′ position of the *bcsAB2‐*N. The *bcs*2 operon also possesses *bcsX* and *bcsY* genes (separated by 421 bp) both described as cellulose production contributing genes.

The *bcs3* cluster contains two ORFs corresponding to a fused cellulose synthase subunits (*bcsAB3*) followed by the *bcsC3* gene (Fig. 3). Other accessory genes related with the cellulose production were absent. It is worth to mention that, in the *K. medellinensis* NBRC 3288, this operon lacks a fragment of 2195 bp that contains the C‐terminal region of *bcsAB3* and the N‐terminal region of *bcsC3*. The version of the *bcsAB3* gene found in the strain NBRC 3288 does not contain a complete BcsB (PF03170) domain, lacking more than the 80% of the sequence compared to the strain *K. medellinensis* ID13488.

Finally, the *bcs4* cluster (Fig. 3) was detected as a stand‐alone copy of the *bcsAB4* gene and, as described in *K. rhaeticus* iGEM (Florea *et al*., 2016a) this nucleotide sequence seems to be more related to *bcsAB2* than the other copies on the genome, suggesting an evolutionary origin in a possible event of duplication of this operon. In addition to this, by comparative genome analysis, a sequence with a 99% identity value to the *bcsAB4* was detected in the chromosome of the non‐producer strain *K. medellinensis* NBRC 3288. The amino acid sequence alignment showed that the product of *bcsAB4* from NBRC 3288 genome differs in 22 amino acids at the N‐terminal domain to that described in the strain ID13488, but its functionality has not been characterized.

### Structural analyses of the *bcs* clusters transcriptional units

To determine the genetic structure and the transcriptional unit composition of the *bcs* clusters found in the strain ID13488 (*bcs1*,* bcs2*,* bcs3* and *bcs4*), retro‐transcription‐PCR assays (RT‐PCR) were performed using total cDNA as template. RNA was isolated from cellulose‐producing cultures of the strain ID13488 grown in static conditions for 8 days (see experimental procedures) and cDNA synthesis was carried out. A set of primers based on the strain ID13488 genomic sequence was designed to cover all genes in each described *bcs* cluster (Tables S2 and S3). RT‐PCR experiments confirmed that all seven genes proposed forming *bcs1* (*bcsZ*,* bcsH*,* bcsA*,* bcsB, bcsC*,* bcsD* and *bglX*) are co‐transcribed in the same polycistronic mRNA and therefore constitute a single operon of 13 795 bp (Fig. 3, S2 and S3). Moreover, it was also confirmed that *bcs1* operon enclosed the 634 bp DNA region upstream of *bcsZ* and the 640 bp DNA region downstream of *bglX* in the transcriptional unit.

The RT‐PCR analysis of the *bcs2* cluster showed an operon constituted of all the five *bcs2* genes, detected by genome sequence analysis plus the genes *rfa*,* kpsC* and *kpsS* located 587 bp upstream of *bcsAB2*‐N. This fact suggests that their transcription is co‐regulated and they could be implied in the cellulose metabolism. The sequencing results also confirmed that the *bcsAB2* gene is divided into two different ORFs of 926 nt (*bcsAB2*‐N) and 2387 nt (*bcsAB2*‐C) in the ID13488 strain (Fig. 3 and S4).

The sequence of the fragments from RT‐PCR analysis also confirmed that the *bcs3* operon consists of the fused *bcsAB3* and the *bcsC3* genes and that the *bcs4* operon is composed of a single fused *bcsAB4* gene (Fig. 3).

Therefore, the results obtained by RT‐PCR analysis prove that *K. medellinensis* ID13488 genome encodes a total of four *bcs* operons related with cellulose biosynthesis and that the *bcs1* operon contains the full set of cellulose synthase genes necessaries to synthetize crystalline BC. However, several differences have been found in gene arrangement of the *bcs2* and *bcs3* operons in comparison with the close related NBCR 3288 strain, as for instance the truncated version of the *bcsAB2* gen in the *bcs2* operon or the deletion of a long DNA fragment affecting to the *bcsAB3* and *bcsC3* genes in the *bcs3* operon.

### Transcriptional analysis of *bcs* operons by quantitative Real‐Time PCR (qRT‐PCR)

**Figure 5:**
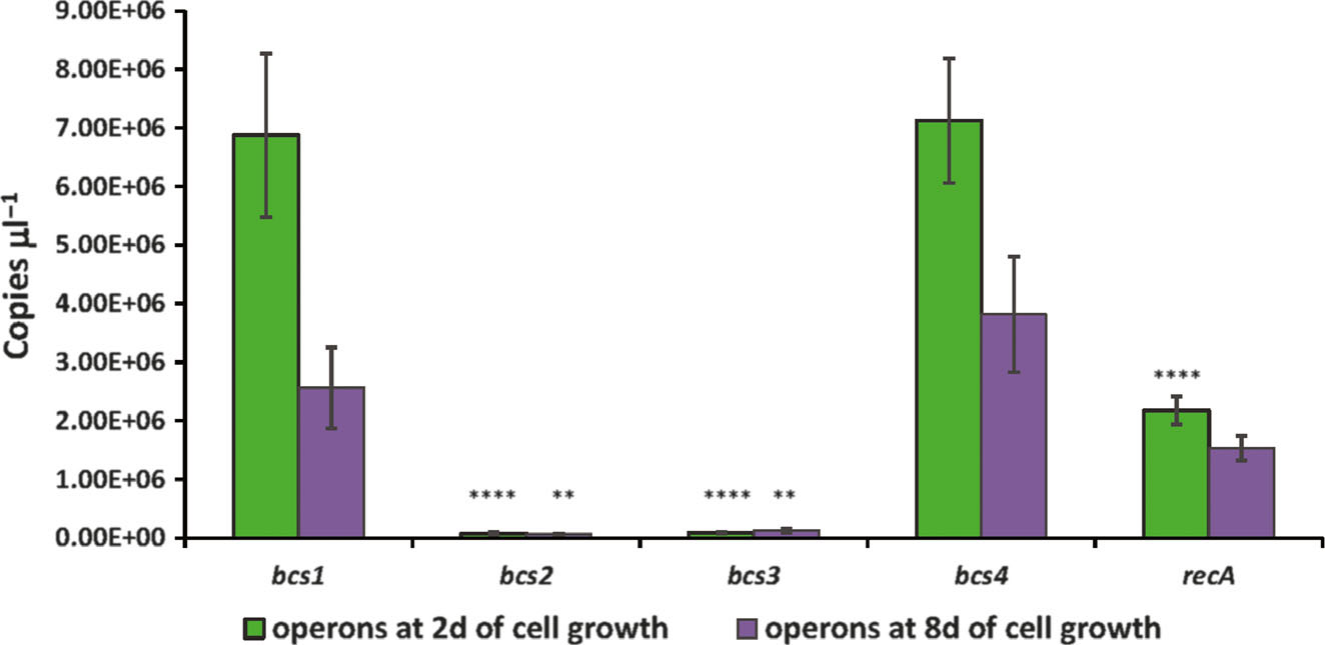
Differential expression of the *bcs* clusters determined by qRT‐PCR. The transcript copy number of the target genes (*bcsA1*,* bcsAB2*,* bcsAB3* and *bcsAB4*) and the control gen, *recA*, was estimated at two time points after culture inoculation (2 or 8 days, d = days), by absolute quantification method. Standard deviations (vertical bars) were obtained from three different assays. Each assay was performed with triplicates of each sample tested. Asterisks mean a significant difference (**P <* *0.01; ***P <* *0.001; ****P <* *0.0001) according to two‐way ANOVA followed by Tukey post‐test to compare with *bcs1* value for 2 days or for 8 days.

Among the four bacterial cellulose synthase operons detected on the strain ID13488, the *bcs1* operon is the only one that contains all the genes required for cellulose biosynthesis and, therefore, seems to be the only one suitable for synthesizing BC type I. To determine the expression profile of each *bcs* operon (*bcs1*,* bcs2*,* bcs3* and *bcs4*), qRT‐PCR assays were carried out at two different time points of bacterial cultivation (2 and 8 days). Total RNA was isolated and reverse transcribed to cDNA. The absolute transcript copy number related to the volume of the sample (copies μl^−1^) of the four *bcs* operons was calculated (see experimental procedures for details). The results showed that the transcripts copy number μl^−1^ calculated for *bcsA* in the *bcs1* and *bcs4* operons after 2 days of cell growth were similar (6.88 × 10^6^ and 7.13 × 10^6^ copies μl^−1^, respectively) and, approximately, 100‐fold higher than the copies calculated for *bcs2* and *bcs3* operons (7.71 × 10^4^ and 8.40 × 10^4^, respectively). Similar copy number values were obtained at 8 days of cell growth (Fig. 5 and Fig. S5). These results suggest that under the conditions assayed, the *bcs1* and *bcs4* operons direct the synthesis of the cellulose in *K. medellinensis* ID13488 whereas the expression of *bcs2* and *bcs3* operons is basal. Further investigation should be carried out to elucidate the function of the *bcs2* and *bcs3* operons. In this sense, the diversity in the regulatory network of cellulose biosynthesis has been discussed by Ryngajllo *et al*. for *Komagataiebacter* strains (Ryngajłło *et al*., 2018), and we cannot exclude that these operons might be active under other environmental growth conditions.

## Conclusions

In this work, we have reported the genomic sequence of the low pH BC producer *K. medellinensis* strain ID13488. We have performed several phylogenetic comparisons and genome sequence comparisons that have shown that the strain ID13488 and the non‐producing BC strain NBRC 3288 are a single species but different strains**,** being the plasmid content and the genetic arrangement of the *bcs1* and *bcs2* operons the most significant differences. The genome of ID13488 codifies four independent *bcs* operons. The synthesis of type I cellulose in the ID13488 strain is driven by the *bcs1* operon, which encodes all the required putative protein products described as required for the nanocellulose production. This operon is transcribed under the BC production conditions at pH 3.6.

## Experimental procedures

### Bacterial strain and growth conditions

*Komagataeibacter medellinensis* strain ID13488 (Castro *et al*., 2013) was purchased from Spanish Type Culture collection (CECT; cat. number 8140). The lyophilized cells were suspended in standard liquid HS medium [(Glucose (20 g l^−1^), yeast extract (5 g l^−1^), citric acid (5 g l^−1^), disodium hydrogen phosphate (2.7 g l^−1^)] all purchased from Sigma Aldrich (St. Louis, MO, USA) and peptone (5 g l^−1^) obtained by Panreac Applychem (Madrid, Spain), at pH 4.0 and cultured statically, at 30°C, for 3 days. The characteristic colony morphology of this strain producing BC was tested by inoculating onto standard HS‐agar plates incubated at 30°C, for 3 days. Cellulose production was also tested by inoculating one single colony into liquid medium. Bacterial cells were maintained as frozen glycerol stocks at −80°C.

For started cultures preparations, standard HS medium (20 ml) was inoculated from glycerol stocks (40 μl) and incubated at 30°C, for 8 days, under static conditions. After this incubation time, BC pellicle was observed floating on the surface of the liquid cultures. When indicated, cellulase [0.3% vol/vol (from *Trichoderma reesei*; C2730; Sigma‐Aldrich)], was added to the cellulose‐producing cultures and then incubated at 30°C, with shaking (180 rpm), for three additional hours.

### DNA and RNA isolations

Cultures of ID13488 strain were grown in the conditions described above [HS standard medium, pH 4.0, with glucose (2%), at 30°C, in static and for 8 days]. Under these growth conditions, the cultures contain a thick matrix of cellulose and most of bacterial cells are attached to the cellulose membrane. To measure the optical density (OD_600 _nm) of these cultures, cellulase (0.2% v/v) was added and incubation with shaking (180 rpm), at 30°C was allowed for three additional hours. In those conditions, cellulose is degraded, and cells are released to the culture media. The OD_600 _nm of these cultures was 0.5 (≈47 × 10^6^ cel ml^−1^). Cultures grown under these conditions were harvested by centrifugation, washed exhaustively with PBS solution and used to nucleic acids (DNA and RNA) isolations.

Genomic DNA was isolated from cellulose‐producing cells using Bacteria Genomic prep MiniSpin Kit (GE Healthcare, Buckinghamshire, UK). Cultures of ID13488 were treated with cellulase and processed as mentioned above. DNA isolation was performed following the instructions from the manufacturer. Integrity of the genomic DNA was checked by gel electrophoresis. Concentration of purified DNA was measured spectrophotometrically (ND1000 spectrophotometer; Thermo Fisher Scientific, Wilmington, NC, USA). For qRT‐PCR assays, genomic DNA concentration was measured fluorometrically (Qubit 2.0 fluorometer; Molecular Probes, California, USA).

Plasmid DNA was isolated from ID13488 cultures grown for 8 days, as have been described above. The cultures were treated with cellulase (0.2% v/v) (C2730; Sigma‐Aldrich, MO, USA) to remove the cellulose matrix. Following, plasmidic DNA was isolated using High Pure Plasmid Isolation Kit (Roche Diagnostics GmbH, Mannheim, Germany). Integrity of the DNA was tested by agarose gel electrophoresis. Purified DNA concentration was measured spectrophotometrically (NanoDrop, Implen GmbH, München, Germany).

Total RNA was isolated from 2 days or for 8 days grown cultures using High Pure RNA Isolation Kit. After this time, cultures were treated with cellulase (0.2% v/v) and both cultures were processed as described above. RNA was obtained following the instructions provided by the manufacturer. After elution, RNA samples were treated with DNase I (Ambion Life technologies, Austin, TX, USA) to avoid genomic DNA contamination in the RNA preparations (see controls in Fig. S2). RNA purity and integrity were tested by measuring the ratio of absorbance 260/280 nm and 260/230 nm and visualized by agarose gel electrophoresis. The amount of total RNA was quantified spectroscopically.

### cDNA Synthesis

Total purified RNA (1 μg) was reverse transcribed using the Transcriptor First Strand cDNA synthesis Kit (Roche Diagnostics GmbH). cDNA synthesis reactions (20 μl) were prepared according to the manufacturer′s instructions and using random hexamers as primers. Before the synthesis reaction, RNA samples were denatured by heating at 65°C for 10 min. Denatured RNA was added to the mix reactions [(transcriptor reverse transcriptase (10 units); reaction buffer (1×); hexamers (60 μM), dNTP mix (1 μM each) and RNase inhibitor (20 units)] and incubated for 10 min at 25°C, 60 min at 50°C and 5 min at 80°C, to inactivate the enzyme. As a control, RT mix reactions were performed without the addition of reverse transcriptase (RT) for each tested condition (Fig. S2).

### Quantitative Reverse Transcription (qRT)‐PCR assays for *bcs* expression analysis

To analyse the expression level of the *bcs* operons in *K. medellinensis* ID13488, qRT‐PCRs assays were performed. Two cultures (50 ml each) inoculated from the same pre‐inoculum (1:500 dilution) were grown for 2 or 8 days in standard HS media and static conditions. Both cultures (2 or 8 days grown) were processed in the same conditions (see above). After RNA isolation and cDNA synthesis, qRT‐PCR was carried out on the LightCycler 480 System (Roche Diagnostics GmbH) and using LightCycler 480 SYBR Green I Master Kit (Roche Diagnostics GmbH). Copy number of the *bcs* operons, in each tested condition, was estimated by absolute quantification method (Whelan *et al*., 2003; Lee *et al*., 2006; Cusick *et al*., 2015).

Amplification reactions (20 μl) were carried out with templates cDNAs diluted 1:20. Sets of primers based on the *bcs*A gene sequence for each operon (*bcs*1, *bcs*2, *bcs3* and *bcs4*) or *recA* control gene were designed using Universal ProbeLibrary System from Roche (Roche Diagnostics GmbH) (Table S4, Fig. S2). qRT‐PCR reaction products ranged from 104 to 112 bp (Fig. S5; Tables S3 and S4). Reactions with each pair of primers were performed by technical triplicates. Each qRT‐PCR experiment was performed in triplicates with the RNA obtained from 2 or 8 days of grown cultures. PCR conditions consisted in one cycle of pre‐incubation at 95°C for 10 min followed by 45 cycles of 5 s at 95°C, 10 s at 60°C and 10 s at 72°C.

Standard curves (SC) were carried out by 10‐fold serial dilution of genomic DNA, ranging from 7.5 × 10^−2^ to 7.5 × 10^−6^ ng μl^−1^ and the *bcsA* primers set specific for each *bcs* operon or *recA* primers set, as control (Table S4, Fig. S5). E values ranged from 1.86 to 1.89 and low variations among the replicates of the *bcsA* genes or *recA* were observed validating the application of the absolute quantification method. The data obtained via SC were used to calculate the transcript copy number of the *bcs1, bcs2, bcs3* and *bcs4* operons after 2 days or 8 days of cell growth. Genome sequence analysis showed that the *bcsA* genes (target genes) and the *recA* gene (control gene) occur as a single copy within ID13488 strain. At the same q‐RTPCR run, cDNAs containing target genes were also amplified with the *bcsA* or *recA* primers sets. The target cDNAs were diluted 1:5 before each assay. Analysis of the raw data was performed with LightCycle 480 SW 1.5 software (Roche Diagnostics GmbH). The threshold cycle (*C*_t_), defined as the PCR cycle at which the fluorescence signal of the SYBR Green dye rises the threshold above the background fluorescence, was calculated for each sample and plotted against the logarithm of the initial DNA concentration. It should be noted that the Ct value obtained in each reaction is inversely related with the amount of the amplicon in the reaction. Specific target gene concentration was obtained based on the linear regression values generated with the SC. Amplification efficiencies values for each primer set were also calculated automatically by the LightCycle software. After thermocycling, a melting curve was made to verify the specificity of the amplified PCR product. The transcript copy number per μl of each target gene was calculated by using absolute quantification method, where:

DNA target concentration (copies μl^−1^) = *N*_A_ × DNA target amount (g μl^−1^)/M_W_ of DNA target
*N*_A _= 6.02 × 10^23^ mol^−1^
*M*_W_ of DNA target* = *DNA target length (dp) × 660 (g mol^−1^ dp^−1^)

### Real‐Time PCR assays (RT‐PCR)

RT‐PCR reactions (25 μl) were carried out using single‐stranded cDNA (2 μl) as template and a set of primers designed for each gene amplification based on the DNA sequence of ID13488 strain (Tables S2 and S3). Reaction mixtures contained Phusion DNA polymerase (1 μl), dNTPs (200 μM) and GC buffer (1×). PCR amplification conditions were tested for each pair of specific primers used. Genomic DNA as template was used as control in all RT‐PCR assays. Size of amplification products was tested by agarose gel electrophoresis (Table S3).

### Genome sequencing and annotation

Genomic DNA from ID13488 strain was extracted from a fresh cells culture, and whole genome DNA was sequencing using PacBio RSII technologies (Pacific Biosciences, Menlo Park, CA, USA) using 10 kb SMRTbell libraries conducted by Macrogen (Macrogen Inc, Seoul, Korea). A total of 142 271 reads were generated with a medium size of 5947 bp. A *de novo* assembly of the sequence was performed also by Macrogen, and a genome draft was obtained organized in three contigs of 2 952 731; 347 611 and 35 852 bp plus a circular sequence of 38 059 bp in size. Additionally, plasmid DNA was sequenced by LifeSequencing (Paterna, Spain) using Illumina (San Diego, CA, USA) MiSeq platform with the Nextera XT 300 × 2 kit. Two extra circular sequences of bp were also identified. After both sequencing processes, a total of 3 381 856 bp with a GC content of 60.6% were assembled. All contigs and plasmids were structurally and functionally annotated using the automated webserver Rast (Aziz *et al*., 2008) with default options. Annotation of plasmids was extended identifying functional domains at Pfam database (Finn *et al*., 2016).

The *bcs* clusters sequences were manually revised and re‐annotated if needed and sequencing errors were also manually. PCR amplifications were also used to confirm and correct the found structural annotation errors. All resulting protein sequences were compared against protein databases performing blastp at Genebank database and HMMER3 (Eddy, 2011) searches, delimiting the putative correct size of the protein and confirming or updating the functional annotation given by Rast.

### Phylogenetic analyses

16S rRNA gene sequences of *K. medellinensis* strain ID13488 were retrieved from the sequenced genome performing local blast searches with *Acetobacteraceae* homolog sequences. Structural annotations of the 16S rRNA were analysed and maintained from those given by the RAST pipeline. All the *Acetobacteraceae* 16S rRNA sequences were retrieved from the Ribosomal Database Project (RDP) (Cole *et al*., 2014) and were aligned using Muscle (Edgar, 2004). The phylogenetic tree was conducted by MEGA 7 (Kumar *et al*., 2016) using the Neighbour–Joining method with bootstrap test values given from 1000 replicates. All the positions containing gaps were eliminated. All ANI values (ANIm) were calculated with the JSpecies software (Richter and Rossello‐Mora, 2009) using MUMmer (Delcher *et al*., 2003) under default conditions.

### *In silico bcs* clusters analyses

To reveal the presence of *bcs* operons in *K. medellinenis* strain ID13488, tblastn searches were performed with the cellulose synthases amino acid sequences encoded by *bcsA* and *bcsB* genes from *G. xylinus* E25 (WP_025437500 and WP_025437501.1) and four putative different operons containing at least one sequence hit were found. To confirm the organization and the annotations of the detected genetic clusters, each gene was compared and if needed, manually re‐annotated. Pairwise blastp was used to compare proteins sequences.

### Sequences

Obtained DNA sequences corresponding to *K. medellinensis* strain ID3488 were deposited at the NCBI database under the QYAZ00000000 accession number.

### Statistical analysis

Statistical analysis was performed by using analysis of variance (ANOVA) followed by Tukey *post hoc* test. GraphPad InStat version 7.0 (GraphPad Software, San Diego, CA, USA) was used for statistical analysis.

## Conflict of interest

None declared.

## Supporting information


**Fig. S1**. Scheme of functional distribution by subsystems using RAST database.

**Fig. S2.** PCR fragments obtained from ID13488 total cDNA amplification.

**Fig. S3**. *K. medellinensis* ID13488 genetic organization of the *bcs1* cluster.

**Fig. S4.** *K. medellinensis* ID13488 genetic organization of the *bcs2* cluster.

**Fig. S5**. Scheme of the four *bcs* clusters analyzed in qRT‐PCR assays. Genetic organization of the clusters analyzed in *K. medellinensis* ID3488.

**Table S1.** ANI values (A) and tetranucleotides signature frequencies correlation coefficients (B) comparison between *K. medellinensis* strain ID13488 and other related strains.

**Table S2**. Primer pairs combinations used to RT‐PCR amplifications. The size of the DNA fragments obtained in each assay is indicated.

**Table S3.** List and DNA sequence of primers used in RT‐PCR amplifications and DNA sequence assays of the *bcs* clusters.

**Table S4.** Primers pairs used for expression analysis performed by qRT‐PCR assays.
