# Supplementary figures and images for: Genome sequence and characterization of the bcs clusters for the production of nanocellulose from the low pH resistant strain Komagataeibacter medellinensis ID13488
Source: Microb Biotechnol. 2019 Feb 22;12(4):620–32. doi: 10.1111/1751-7915.13376 (PMC6559206; doi:10.1111/1751-7915.13376)

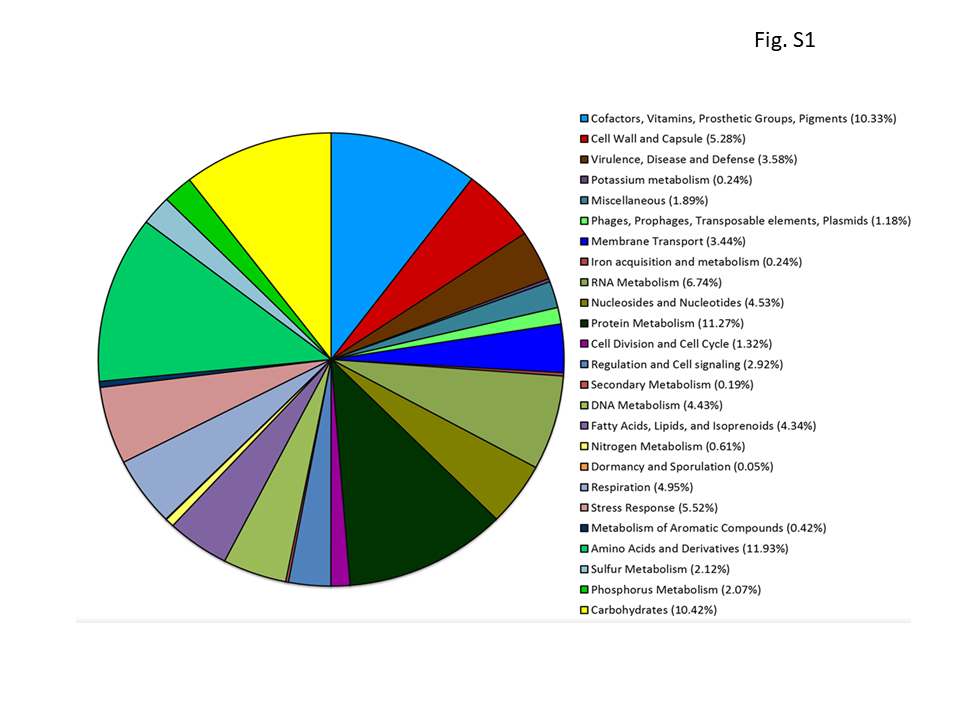

Supplement: Supplementary file 1 — Fig. S1. Scheme of functional distribution by subsystems using RAST database. [file MBT2-12-620-s001.tif]

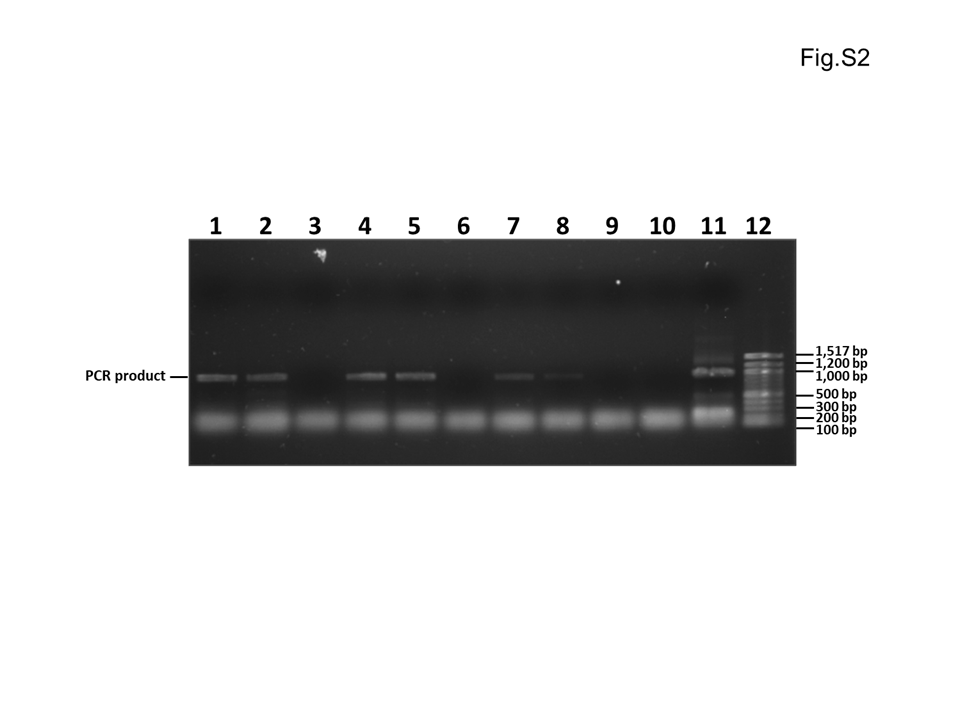

Supplement: Supplementary file 2 — Fig. S2. PCR fragments obtained from ID13488 total cDNA amplification. [file MBT2-12-620-s002.tif]

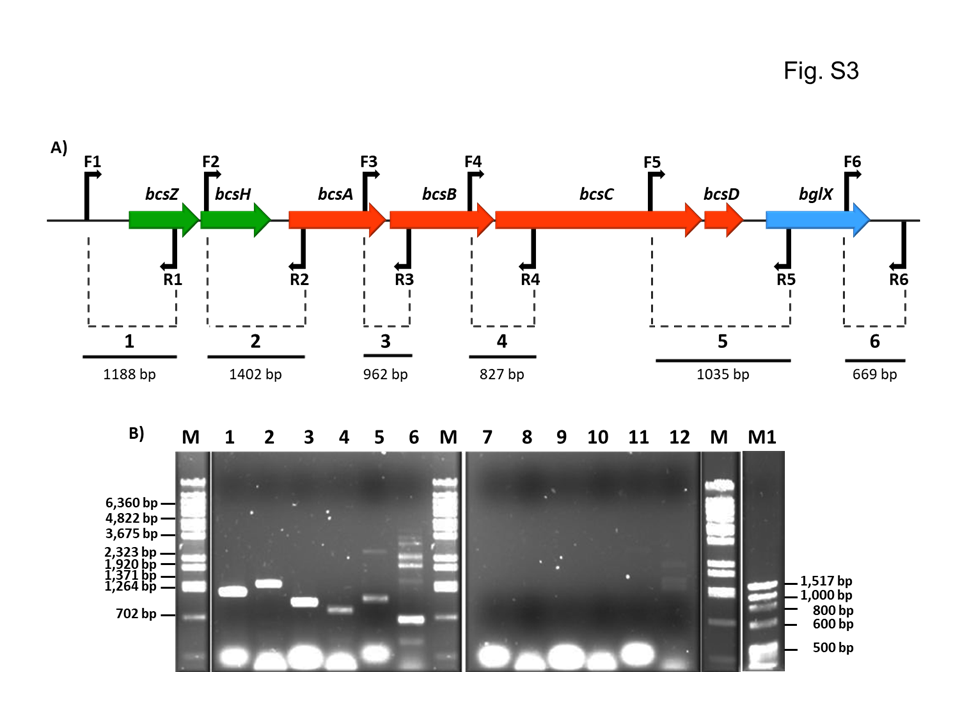

Supplement: Supplementary file 3 — Fig. S3. K. medellinensis ID13488 genetic organization of the bcs1 cluster. [file MBT2-12-620-s003.tif]

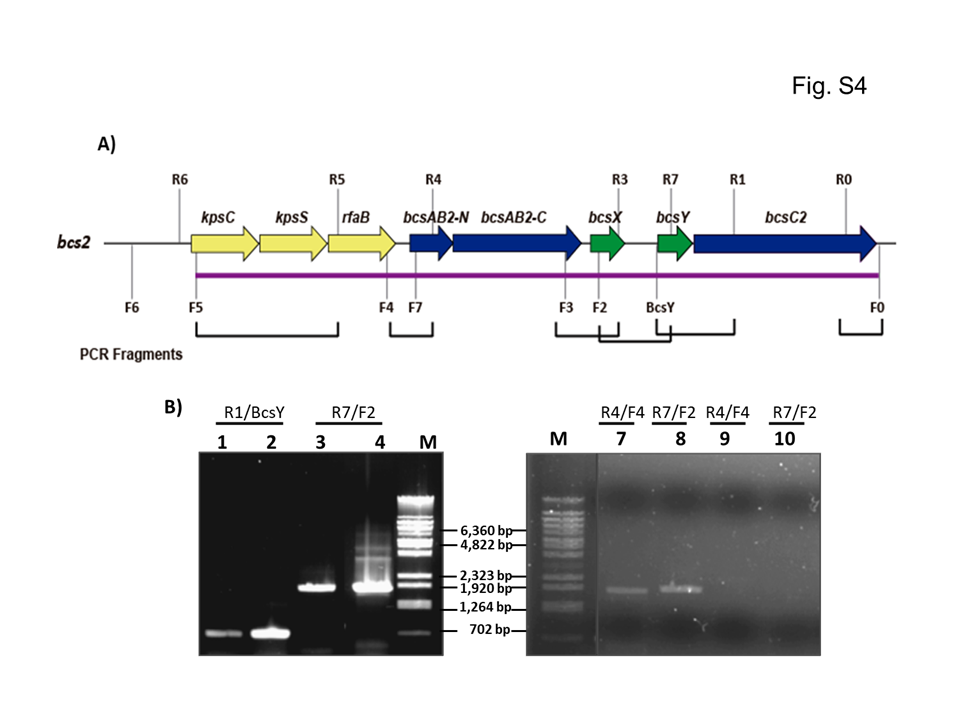

Supplement: Supplementary file 4 — Fig. S4. K. medellinensis ID13488 genetic organization of the bcs2 cluster. [file MBT2-12-620-s004.tif]

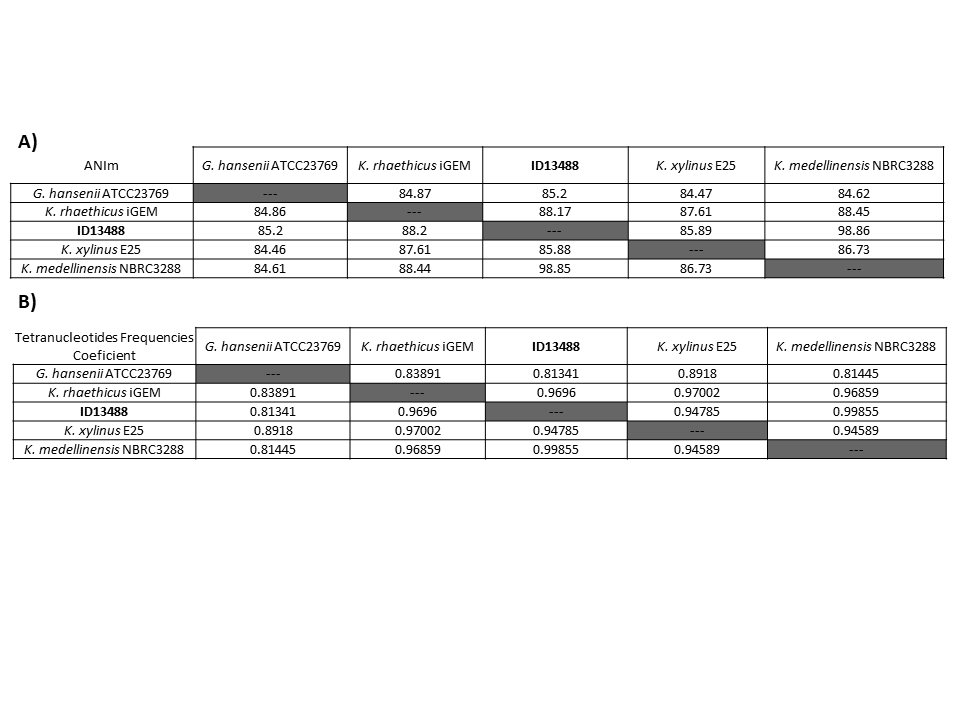

Supplement: Supplementary file 6 — Table S1. ANI values (A) and tetranucleotides signature frequencies correlation coefficients (B) comparison between K. medellinensis strain ID13488 and other related strains. [file MBT2-12-620-s006.tif]
