# Supplementary material for: Genome sequence and characterization of the bcs clusters for the production of nanocellulose from the low pH resistant strain Komagataeibacter medellinensis ID13488
Source: Microb Biotechnol. 2019 Feb 22;12(4):620–32. doi: 10.1111/1751-7915.13376 (PMC6559206; doi:10.1111/1751-7915.13376)
Supplement: Supplementary file 7 — Table S2. Primer pairs combinations used to RT‐PCR amplifications. The size of the DNA fragments obtained in each assay is indicated. [file MBT2-12-620-s007.docx]

**Table S2.** Primer pairs combinations used to RT-PCR amplifications assays.

| **Operon** | **Primer pairs** | **Fragment size (bp)** |
| --- | --- | --- |
| ***bcs1*** | F3C1/R3C1 | 1188 |
|  | F1C1/R8C1 | 1402 |
|  | F9C1/R9C1 | 962 |
|  | F12C1/R12C1 | 827 |
|  | F4C1/R7C1 | 1035 |
|  | F4C1/R4C1 | 605 |
|  | F4C1/R5C1 | 1872 |
|  | F3C1/R2C1 | 1391 |
|  | F2C1/R2C1 | 1091 |
|  | F5C1/R5C1 | 1361 |
|  | F2C1/R1C1 | 1882 |
|  | F5C1/R6C1 | 2469 |
|  | F6C1/R6C1 | 669 |
|  | F13C1/R13C1 | 1290 |
|  | F14C1/R13C1 | 1370 |
|  | F15C1/R13C1 | 1488 |
|  | F6C1/R14C1 | 1090 |
|  | F13C1/R13C1 | 1792 |
|  | F16C1/R13C1 | 1609 |
|  | F17C1/R13C1 | 1702 |
|  | F18C1/R13C1 | 1792 |
|  | F6C1/R14C1 | 1094 |
|  |  |  |
| ***bcs2*** | R4C2/F4C2 | 1727 |
|  | R3C2/F3C2 | 1098 |
|  | R7C2/F7C2 | 1804 |
|  | R0C2/F0C2 | 923 |
|  | R5C2/F5C2 | 1428 |
|  | R6C2/F6C2 | 980 |
|  | R7C2/F2C2 | 1804 |
|  | R1C2/F2C2 | 2327 |
|  | BcsY/R1C1 | 671 |
|  |  |  |
| ***bcs3*** | F1´C3/R1´C3 | 794 |
|  | F1C3/R1C3 | 1028 |
|  | F2C3/R2C3 | 754 |
|  | F3C3/R3C3 | 680 |
|  | F3C3/R3´C3 | 848 |
|  | F5C3/R5C3 | 1301 |
|  |  |  |
| ***bcs4*** | F0C4/R0C4 | 997 |
|  | F1C4/R1C4 | 1288 |
|  | F2C4/R2C4 | 1004 |
|  | F3C4/R3C4 | 1059 |
|  |  |  |
