# Supplementary material for: Genome sequence and characterization of the bcs clusters for the production of nanocellulose from the low pH resistant strain Komagataeibacter medellinensis ID13488
Source: Microb Biotechnol. 2019 Feb 22;12(4):620–32. doi: 10.1111/1751-7915.13376 (PMC6559206; doi:10.1111/1751-7915.13376)
Supplement: Supplementary file 8 — Table S3. List and DNA sequence of primers used in RT‐PCR amplifications and DNA sequence assays of the bcs clusters. [file MBT2-12-620-s008.docx]

**Table S3.** Primers used in RT-PCR amplification and DNA sequence assays of the *bcs* clusters.

| **Name** | **Sequence (5´- 3’)** | **Genome** | **Operon** |
| --- | --- | --- | --- |
| F1C1 | ACGTGGCCCGTATGCTGACCGGTGGCTATGGAC | NBRC 3288 | *bcs1* |
| F2C1 | TCGATACCGGCAATAGTGGTGAGTCCCATAGTGAAGGGC | NBR C 3288 | *bcs1* |
| F3C1 | CTTTATCTTGCAGGATGAAGGAGTTTGCGATTA | NBRC 3288 | *bcs1* |
| F4C1 | GTCTTACTGGCGGATTTAATGCTAGGGTCGGGTATCGATTTA | NBRC 3288 | *bcs1* |
| F5C1 | GAGACTGTCCCGCAAGATATGCCTGCTTTCCGCCG | NBRC 3288 | *bcs1* |
| F6C1 | CAATCACCTCAAGCCGCTCTACCCGTTCGGGT | NBRC 3288 | *bcs1* |
| F9C1 | GGTGGTGTTCTGGAGAACGAGGAAT | CECT | *bcs1* |
| F12C1 | TCTCGCCCTGCTTTCGGCTGACG | CECT | *bcs1* |
| F13C1 | TCTTGCGTTCCGGTTTGTTGGTGTGT | CECT | *bcs1* |
| F14C1 | ATCCTTTGAATTTCTGCAAGAATTATCCCCG | CECT | *bcs1* |
| F15C1 | TTGCCGTCCGTCTAAAAACATTGATT | CECT | *bcs1* |
| F16C1 | TCAAGGCGGGTTTTTCGTATGTAATCAGG | CECT | *bcs1* |
| F17C1 | AGGACGCCGCCCTCTCAC | CECT | *bcs1* |
| F18C1 | CGGGAAGGGGGAGTGATTATCTC | CECT | *bcs1* |
|  |  |  |  |
| R1C1 | TCAGGATTGCCCGTTTTCTTCCTCGCGCTTGCGTAACAGG | NBRC 3288 | *bcs1* |
| R2C1 | TGGTGCCGTGTGTCATCCTCGTAACCAGAGGGTAGATCCG | NBRC 3288 | *bcs1* |
| R3C1 | TCCCTCGCCACGGGCAATATAAACCAGCAATGTCAGGGC | NBRC 3288 | *bcs1* |
| R4 C1 | TGCTGCGGGTGCGCATGTACAGAATGATCG | NBRC 3288 | *bcs1* |
| R5 C1 | CGGATGGCTGACCAGCCCTGCCGCAAACAG | NBRC 3288 | *bcs1* |
| R6 C1 | CGGCAGGGGGCGCATCGTCAGGCTCGGCCA | NBRC 3288 | *bcs1* |
| R7 C1 | ATTGAAACCGCCGCCATCCACGCTGAACAG | NBRC 3288 | *bcs1* |
| R8 C1 | CAGCGTTTCAGTCAGACGCCATGTCAGGTAGCGCAGCGA | CECT | *bcs1* |
| R9 C1 | TCCGCATTGTCCACAATGGTATTAACCGCGCT | CECT | *bcs1* |
| R12 C1 | TGGCGGCTTACCACCACTGAACAGC | CECT | *bcs1* |
| R13C1 | TCCCTCGCCACGGGCAATATAGACCAGCAATGTCAGGGC | CECT | *bcs1* |
| R14C1 | TCAAGCAATGTTGTTGCGTAAAAA ATCCCAA | CECT | *bcs1* |

| **Name** | **Sequence (5´- 3’)** | **Genome** | **Operon** |
| --- | --- | --- | --- |
| F0C2 | GCT TTC TGT TGC AGA TCA GCC GGA | CECT | *bcs2* |
| F2C2 | CGC GGG TCT TAC GCT TCT GAT TAT CGG | CECT | *bcs2* |
| F3C2 | GCC CGC TCC ATC ATT GCC TTT ACG | CECT | *bcs2* |
| F4C2 | ACC CCT TTT GTT TCA GTG GCC GGA GC | CECT | *bcs2* |
| F5C2 | CGT ATT CGC CAG AAT GCA TGG GCC | CECT | *bcs2* |
| F6C2 | TGG GGA AGG ACA GGA GTG TCA TGC | CECT | *bcs2* |
| F7C2 | GTA GCC GCC AGA ATT ACC GAA TTG ATG | CECT | *bcs2* |
| F-BcsY | GCA TTG TGG TGG CGA GCT ATC TTC TG | CECT | *bcs2* |
|  |  |  |  |
| R0C2 | GGT GAA GGT TGC GGG TAA TGG CT | CECT | *bcs2* |
| R1C2 | GGC TTC ATT GTT GTC CGG CTC G | CECT | *bcs2* |
| R3C2 | CCG CAG GAG CTG CTT TCA TCT TCT | CECT | *bcs2* |
| R4C2 | CCA CCC TCT GCG CTG CAT CTT GAG | CECT | *bcs2* |
| R5C2 | CGG GCA ATC AGA CGT GAA AGG TCC | CECT | *bcs2* |
| R6C2 | CAG CAG GGC ATG CGC AAC CAT ATT CC | CECT | *bcs2* |
| R7C2 | GCAGGCGTCACCCGGTCT TCG T | CECT | *bcs2* |

| **Name** | **Sequence (5´- 3’)** | **Genome** | **Operon** |
| --- | --- | --- | --- |
| F1C3 | ATG ATC TGG CGT ATT TTA AAG TCC CCC C | CECT | *bcs3* |
| F1´C3 | ATG CGA GGG AAC ATG GAA TTT CC | CECT | *bcs3* |
| F2C3 | CAC GGG CGG GAT CTA CAC GAT GGG C | CECT | *bcs3* |
| F3C3 | CGG GAT CGA GCT TAC GCC GCA CC | CECT | *bcs3* |
| F5C3 | CTA TCA ACC GCT ATC CCG TCA CCG | CECT | *bcs3* |
|  |  |  |  |
| R1C3 | GTT TCC GTC TCC AGC CCT GCC GC | CECT | *bcs3* |
| R1´C3 | CAA GGG GGG ACT TTA AAA TAC GCC AG | CECT | *bcs3* |
| R2C3 | GAC CGT GTG CTG TCC GGC TCC AT | CECT | *bcs3* |
| R3C3 | TGC CAT GCA CCT CAT CCC AAC TA | CECT | *bcs3* |
| R3´C3 | GCA AGC TGG TCC ACA CGC CCT GA | CECT | *bcs3* |
| R5C3 | CCA TGA CTG AAC CGT TTC ATC GCG | CECT | *bcs3* |
| R6C3 | CGC ACC CTG TAT GTA TCG ACA ATG C | CECT | *bcs3* |

| **Name** | **Sequence (5´- 3’)** | **Genome** | **Operon** |
| --- | --- | --- | --- |
| F0C4 | TGC TGG GTG GCG GTG GTC GG | CECT | *bcs4* |
| F1C4 | ACA AAG CCG CTG GTG TCA CTG C | CECT | *bcs4* |
| F2C4 | GTG GCC TGC GTA ATG GTG TAA GCG | CECT | *bcs4* |
| F3C4 | TCA GAT TGG GAT GCC AGC GAA ACC | CECT | *bcs4* |
|  |  |  |  |
| R0C4 | TCA GGC ACA GGG TGG AGA GCG CA | CECT | *bcs4* |
| R1C4 | AAA TGC GAC ATG GCC GAC AGG T | CECT | *bcs4* |
| R2C4 | CGG TTG GGG TCG TGT GCA GAT G | CECT | *bcs4* |
| R3C4 | CAC CCC AAT GTT CGT CGC GGA | CECT | *bcs4* |
